# Can AI Capture and Quantify Clinical Expertise? Implications for Intracoronary Imaging in Percutaneous Coronary Intervention

**DOI:** 10.1016/j.jscai.2024.102523

**Published:** 2025-03-18

**Authors:** Ricardo Petraco, Rahul Bahl, Guilherme Almeida, Daniel Bandeira, Henry Seligman, Anissa Alloula, Abigail Demuyakor, Alexandra J. Lansky, Tom Johnson, Darrel Francis, Gary S. Mintz, Matthew Shun-Shin, Daniel Chamié

**Affiliations:** a Department of Cardiology, Imperial College Healthcare NHS Trust, London, United Kingdom; b London Core Laboratory, London, United Kingdom; c National Heart and Lung Institute, Imperial College London, London, United Kingdom; d Department of Medicine, Federal Institute of Sergipe, Sergipe, Brazil; e Section of Cardiovascular Medicine, Department of Internal Medicine, Yale School of Medicine, New Haven, Connecticut; f Big Data Institute, University of Oxford, Oxford, United Kingdom; g Bristol Heart Institute, University of Bristol, Bristol, United Kingdom; h Cardiovascular Research Foundation, New York, New York

**Keywords:** artificial intelligence, coronary artery disease, intravascular imaging, intravascular ultrasound, optical coherence tomography

With recent international guidelines updates placing the use of intravascular imaging (IVI) as a class I indication for percutaneous coronary intervention (PCI) guidance,^1^ it is reasonable to expect that the adoption of modalities such as intravascular ultrasound (IVUS) and optical coherence tomography (OCT) will increase significantly over the next few years. IVI use is beneficial over angiography alone as it allows for a more comprehensive assessment of disease burden, precise vessel measurements, and stent sizing, thereby preventing the complications associated with a geographic miss and stent underexpansion.^2^^,^^3^ Many physicians who currently do not use IVI will start doing so, and those who already use it will likely increase their volume. Such a surge in IVI utilization globally needs to be coupled with increased education, training, and improvements in technology, with a focus on facilitating user interface and procedural guidance. Artificial intelligence (AI)-based software will come top on the list of tools that will enable such transformations at a fast pace.

Over the last 20 years, research on the application of AI in coronary imaging has increased exponentially. A brief PubMed search for peer-reviewed research on AI and IVI, limited to those published since 2000, reveals an exponentially expanding field. We identified 195 papers, with only 1 paper published in 2005 and 47 in 2023. Although many of the early studies focused on image optimization and reconstruction,^4^, ^5^, ^6^ more recent models have a much broader range of considerations, including the prediction of patient and procedural outcomes, plaque phenotyping and vulnerability, and the characterization of new biomarkers of coronary artery disease.^7^, ^8^, ^9^

In this issue of *JSCAI*, Shin et al^10^ offer a comprehensive review of the current IVUS and OCT technologies that use AI-capable software in clinical consoles. Although user interfaces and displays vary among vendors, they all offer similar capabilities focused on tracking vessel layers and tissue characterization*.* It is now possible to automatically identify vessel lumen and media (or external elastic membrane when applicable) and trace them with minimal input from the operator. This allows for increased precision and speed in vessel measurements during PCI, which permits operators to make decisions on stent sizing much more reproducibly and, potentially, to lower the risk of under or oversizing. Second, current AI tools can identify coronary artery calcium—the lesion morphology commonly associated with stent underexpansion—with increased precision. AI-derived calcium detection assists physicians with less imaging experience in assessing calcific burden, which has been shown to predict stent underexpansion and thereby highlight lesions that may benefit from advanced lesion preparation during PCI.^11^

Current AI approaches for vessel layer tracking and tissue characterization have a few similarities in their benefits and limitations to clinical use. First, the process of building an AI network training for tasks such as tracking the vessel lumen or identifying a calcific segment on a cross-sectional image is relatively simple to implement hence, we should expect an increase in modalities that offer the same output. For instance, AI-derived border tracking is widely used in other areas of medical imaging, including cardiac imaging, such as echocardiography, computed tomography, and magnetic resonance imaging. Second, the process of training the AI system for such tasks can be undertaken by individuals with basic to intermediate knowledge of coronary interventions, such as engineers, clinical scientists, and other allied professionals. The principle of easy accessibility to AI network trainers facilitates the development of AI-based software, as such labor-intensive work does not need to be undertaken by physicians with large expertise in coronary intervention or IVI interpretation. Over the next few years, we should see substantial improvements in AI-based tools to increase the accuracy of coronary vessel tracing and tissue identification as the systems continue to learn.

## Capturing expert judgment with AI for imaging-guided PCI

Currently, available AI software have limitations with respect to the type of hierarchical intelligence they can offer. Although they can help with questions such as “where is the vessel and what is its size?” or “where is the calcium in this vessel?” they still fail to go a step further and answer “can I trust the imaging quality of this OCT run?” or “where should I land the stent in such a diseased calcified vessel?” During PCI, operators instinctively capture interconnected pieces of imaging-based information and make nuanced decisions based on expert intelligence developed over years of practice. Therefore, for AI systems to help answer questions of increased clinical complexity, they must learn to *capture clinical expertise* and output it in the form of usable indices. A unique collaboration between our research groups from Imperial College London, Yale School of Medicine, and London Core Laboratory is developing such AI-based tools to apply to IVI. We present here the prototype of 2 AI-based OCT software being developed to capture expert intelligence to aid physicians in PCI decision-making.

## “Is this OCT image of good enough quality to be used for decision-making?”

**Figure 1:**
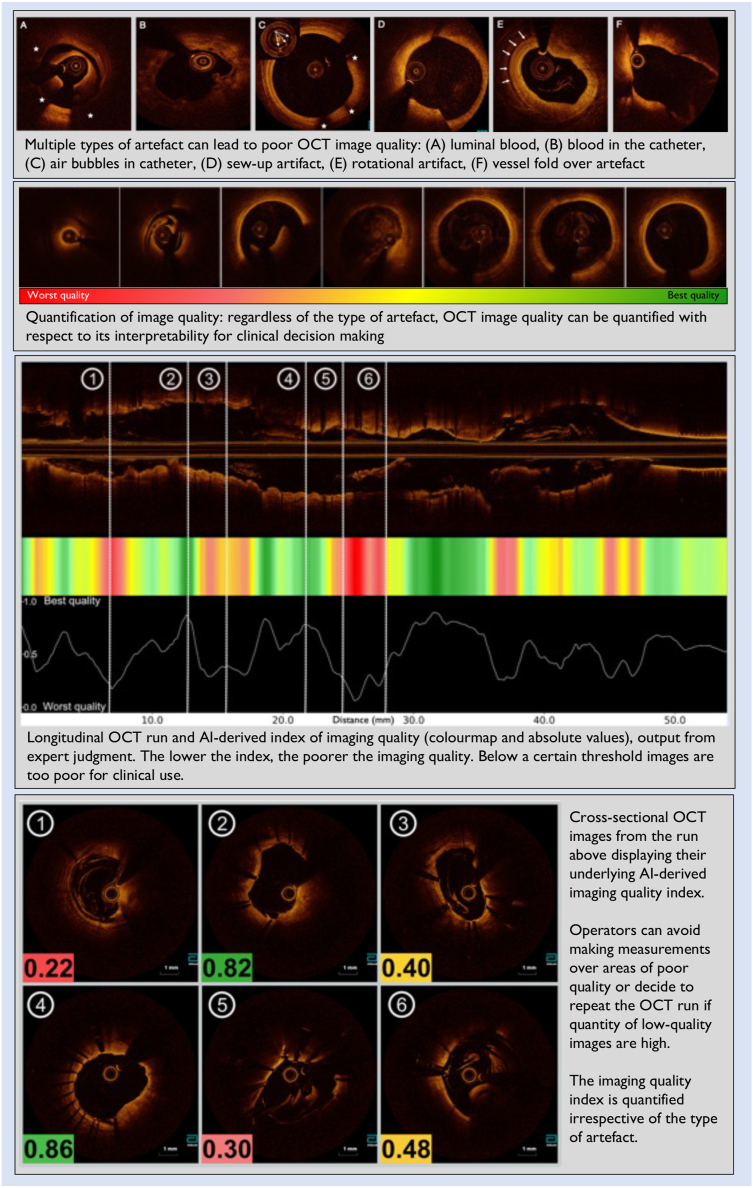
**Artificial intelligence–based tool to quantify optical coherence tomography imaging quality**.

Several types of artifacts can preclude optimal OCT imaging generation, which is the first and fundamental step for accurate PCI guidance. These include poor blood clearance (from lumen and catheter), nonuniform rotational distortions, sew-up, ghost lines, and vessel fold-over (upper panel, Figure 1). Artifacts often coexist in the same image and affect the image quality on a continuum, ranging from very mild and insignificant to severely poor and uninterpretable (second panel, Figure 1). It is up to the PCI operator to decide whether the OCT image is of sufficient quality for decision-making, regardless of the type of artifact present. Based on the above rationale, we have developed an AI-assisted software that quantifies OCT image quality and outputs it as an index (bottom panels, Figure 1). Importantly, our AI tool is not based on any specific technical criteria such as the quantity of luminal blood or magnitude of image distortion, but instead on expert judgment of image quality. This tool could aid PCI guidance in several ways. First, it could prevent physicians from trying to make measurements at segments where image quality is below a certain standard, which could lead to stent sizing errors. Second, this could prompt physicians to repeat the OCT run in cases where the overall burden of poor-quality images is high, potentially fixing the initial error that led to the artifact.

## “Where should I land the stent?”

Identification of adequate landing zones is a critical step of the pre-PCI procedural planning. Ideal landing zones should be where the vessel is as close as possible to normal and have the largest lumen.^12^^,^^13^ Identifying optimal landing zones allows for safe positioning of the stent edges, ensuring complete lesion coverage and optimal stent expansion without edge complications. The proximal and distal landing zones determine the length and diameter of the stent(s) to be implanted and the diameter of the balloons to predilate the lesion and postdilate the stent(s). Conversely, landing the stents in regions with significant plaque burden (>50%) is associated with an increased risk of stent edge restenosis.^13^^,^^14^ Furthermore, landing stent edges on a lipidic plaque, specifically on a thin-cap fibroatheroma, can increase the risks of edge dissections,^15^^,^^16^ distal embolization,^12^ and early and late target lesion failure.^13^^,^^17^, ^18^, ^19^ Incomplete lesion coverage with significant reference vessel plaque and lumen area <4.5 mm^2^ at either the distal or proximal stent edges have also been identified as independent predictors of stent failure.^20^

Current OCT systems provide a lumen profile that incorporates a multiplanar reconstruction of 3-dimensional lumen dimensions, which allows for easier and immediate identification of the lumen stenoses and adjacent regions with the largest lumen dimensions that could serve as landing zones. More recently, most OCT systems incorporate AI-assisted detection of the external elastic membrane onto the lumen profile, enhancing the identification of the most normal-looking reference segments. Although these features represent significant advancements in IVI guidance, it is still up to the operator to decide on landing zone selection based on a broader range of imaging nuances (underlying plaque type, disease distribution and extension, presence, and size of side branches, luminal thrombus, previous stents, dissections, etc). Although this might seem straightforward in less complex focal lesions surrounded by near-normal references, the selection of landing zones can be more challenging in situations of diffuse disease, where one must land 1 or both stent edges on the plaque. In some cases, multiple potential landing zones exist, and one needs to decide between a short (focal) or a long stent or multiple stents. Finally, operators who have less experience with IVI interpretation might find it more challenging to make landing zone decisions when compared to IVI experts.

**Figure 2:**
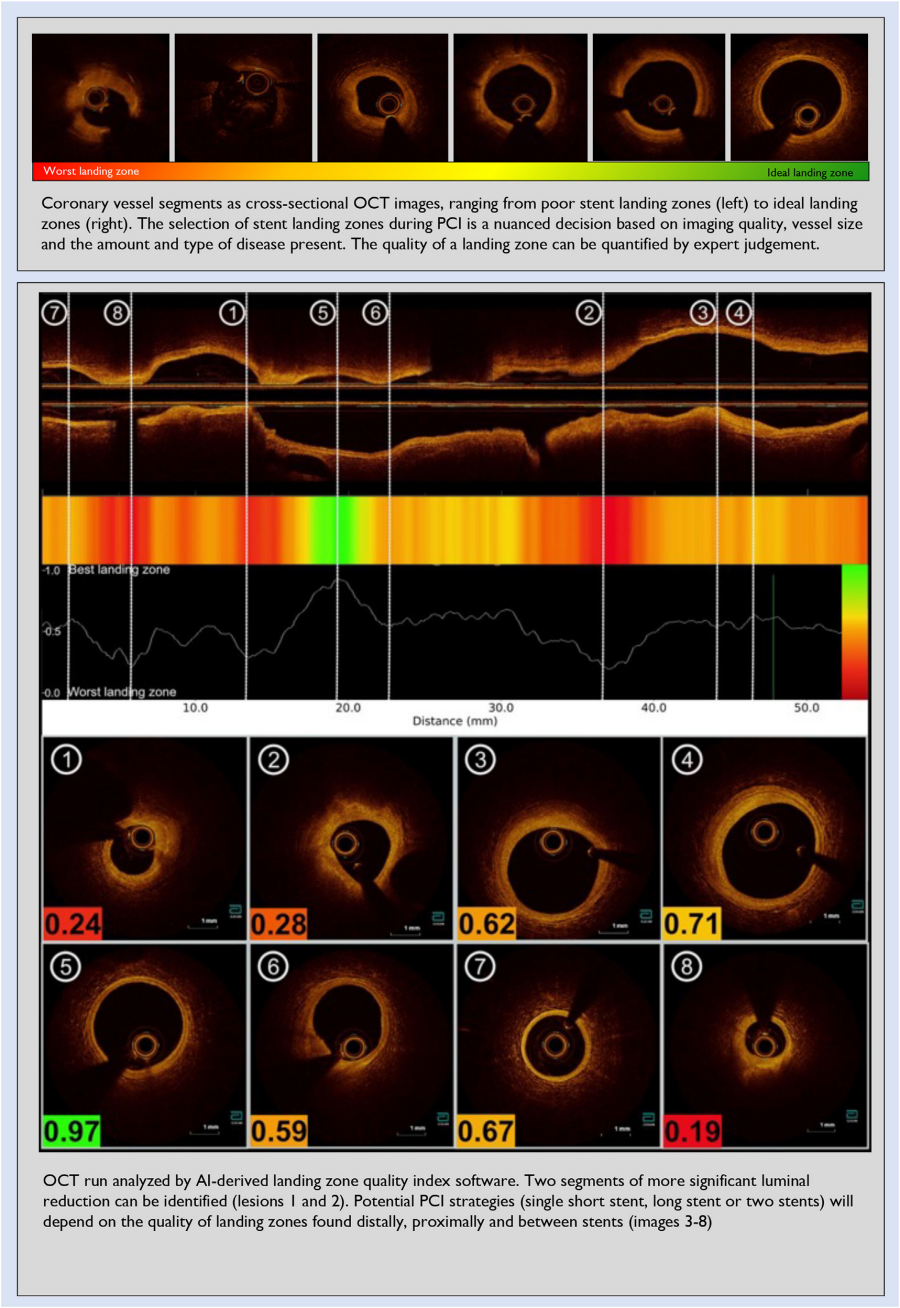
**Artificial intelligence–based tool to quantify the quality of stent landing zones on optical coherence tomography**.

In Figure 2, we present an OCT run analyzed with our AI-based landing zone detection software, where the quality of landing zones can be quantified and displayed as a quantifiable index. Equally, this index was built by capturing overall expert judgment and not based on specific imaging criteria related to tissue characterization, plaque burden, or any other technical metric.

## Conclusions and future directions

The process of capturing expert gestalt and transforming it into clinically applicable tools usable by other physicians is only possible with modern AI training and could potentially offer a paradigm shift in the way imaging guidance is provided and interpreted. AI systems trained by expert physicians allow for the first time for their brains to be part of the building blocks of imaging software (and not only engineers and coders), providing less experienced operators greater confidence in decision-making requiring advanced image interpretation. Many more relevant clinical questions could be addressed with similar AI approaches applied to both OCT and IVUS modalities, including assessment of stent expansion and the need for calcium modification before stent deployment. This also opens the opportunity for potential new trials testing the safety of AI vs physician-guided PCI. We are only at the very start of an era of AI-assisted IVI usage and are yet to fully appreciate the scope and impact of machine learning on PCI decision-making.
